# Rotavirus and adenovirus infections in children with acute gastroenteritis after introducing the Rotasiil^®^ vaccine in Kisangani, Democratic Republic of the Congo

**DOI:** 10.1371/journal.pone.0297219

**Published:** 2024-02-12

**Authors:** Didier Gbebangi Manzemu, Jean Pierre Alworong’a Opara, Emmanuel Tebandite Kasai, Mupenzi Mumbere, Véronique Muyobela Kampunzu, Bibi Batoko Likele, Naura Apio Uvoya, Hortense Malikidogo Vanzwa, Gaspard Mande Bukaka, Falay Sadiki Dady, Nestor Ngbonda Dauly, Laurent Belec, Serge Tonen-Wolyec

**Affiliations:** 1 Department of Pediatrics, Faculty of Medicine and Pharmacy, University of Kisangani, Kisangani, Democratic Republic of the Congo; 2 Department of Pediatrics, Faculty of Medicine, Catholic University of the Graben, Butembo, Democratic Republic of the Congo; 3 Department of Pediatrics, Faculty of Medicine, University of Bunia, Bunia, Democratic Republic of the Congo; 4 Laboratoire de Virologie, Hôpital Européen Georges Pompidou, Assistance Publique-Hôpitaux de Paris, and University of Paris, Sorbonne Paris Cité, Paris, France; 5 Department of Internal Medicine, Faculty of Medicine, University of Bunia, Bunia, Democratic Republic of the Congo; 6 Department of Internal Medicine, Faculty of Medicine and Pharmacy, University of Kisangani, Kisangani, Democratic Republic of the Congo; Universita degli Studi di Parma, ITALY

## Abstract

**Background:**

Although rotavirus vaccination has reduced the global burden of the virus, morbidity and mortality from rotavirus infection remain high in Sub-Saharan Africa. This study aimed to determine the prevalence of rotavirus and adenovirus infections in children under five years with acute gastroenteritis and to identify factors associated with rotavirus infection after the introduction of the Rotasiil^®^ vaccine in 2019 in Kisangani, Democratic Republic of the Congo (DRC).

**Methods:**

This study consisted of a cross-sectional hospital-based survey conducted from May 2022 to April 2023 in four health facilities in Kisangani, using a fecal-based test (rapid antigenic immuno-chromatographic diagnostic test, BYOSYNEX adenovirus/rotavirus BSS, Biosynex SA, Illkirch-Graffenstaden, France) of rotavirus and adenovirus infections among children under five years of age with acute gastroenteritis.

**Results:**

A total of 320 children under five years of age with acute gastroenteritis were included. The prevalence of rotavirus infection was 34.4%, that of adenovirus was 6.3%, and that of both rotavirus and adenovirus coinfection was 1.3%. The prevalence of rotavirus was significantly higher in unvaccinated children than in vaccinated children (55.4% versus 23.1%; P < 0.001). This difference was observed only in children who received all three vaccine doses. Multivariate logistic regression analysis shows that the rate of rotavirus infection was significantly reduced in vaccinated children (adjusted OR: 0.31 [95% confidence intervals (CI): 0.19–0.56]; P < 0.001) and those whose mothers had an average (adjusted OR: 0.51 [95% CI: 0.25–0.91]; P = 0.018) or high level (adjusted OR: 0.34 [95% CI: 0.20–0.64]; P < 0.001) of knowledge about the rotavirus vaccine.

**Conclusions:**

The prevalence of rotavirus infection remains high in Kisangani despite vaccination. However, the prevalence of adenovirus infections was low in our series. Complete vaccination with three doses and mothers’ average and high level of knowledge about the rotavirus vaccine significantly reduces the rate of rotavirus infection. It is, therefore, essential to strengthen the mothers’ health education, continue with the Rotasiil^®^ vaccine, and ensure epidemiological surveillance of rotavirus infection.

## Introduction

Acute gastroenteritis caused by viruses is one of the leading causes of mortality in children under five years of age in developing countries [1, 2]. Among these enteric viruses, rotavirus is the most prevalent and lethal worldwide [2]. Indeed, rotavirus is responsible for destroying mature enterocytes, thereby reducing the absorption capacity of the villi and causing diarrhea [3, 4]. Therefore, the spectrum of rotavirus gastroenteritis ranges from transient stool softening to severe diarrhea and vomiting, which can be complicated by severe dehydration and death [5]. Before rotavirus vaccines became available in 2006, rotavirus was associated with over 500,000 deaths and more than two million hospitalizations worldwide [5–7]. This statistic motivated the implementation of effective interventions, such as rotavirus vaccination early in life to reduce the global burden of severe rotavirus gastroenteritis [5].

Currently, four rotavirus vaccines prequalified by the World Health Organization (WHO) are available for routine vaccination. The first two were the RotaTeq (Merck & Co. Inc., Whitehouse Station, NJ, USA) in 2008 and the Rotarix (GlaxoSmithKline Biologicals, Rixensart, Belgium) in 2009. The other two vaccines prequalified by the WHO in 2018 were Rotavac (Bharat Biotech International Ltd, India) and the Rotasiil^®^ (Serum Institute of India, India) [5]. The Rotasiil^®^ vaccine, a pentavalent vaccine targeting genotypes frequently implicated in rotavirus infection in sub-Saharan Africa (SSA), was introduced in 2019 in most African vaccination programs for children under 11 months [8, 9]. The Rotasiil^®^ vaccine is administered orally, and three doses at four-week intervals are recommended for best protection, with the first dose given at six weeks of age [9].

The Democratic Republic of the Congo (DRC) is one of four countries, including India, Nigeria, and Pakistan, that account for almost half of all rotavirus-related deaths [7]. In this country, the rate of rotavirus infection was 60% in children under five years of age admitted with acute gastroenteritis before the vaccine’s introduction in 2019 [10, 11]. To the best of our knowledge, empirical findings on the epidemiology and clinic of rotavirus are scarce, apart from a few results from sentinel rotavirus post-vaccination surveillance sites in the DRC. At the same time, clinicians continue to deal with this infectious disease in their daily practice. The WHO reports that rotavirus continues to kill despite vaccination, with a mortality rate of 33 deaths per 100,000 children under the age of five years [5]. A systematic review by Lamberti et al. reported that rotavirus vaccination efficacy was 90% in developed countries, 88% in East and Southeast Asia, and only 46% in SSA [12]. Because the vaccine has not yet controlled rotavirus gastroenteritis, and the fact that less effective vaccines could have a considerable impact on reducing the burden of disease in high-prevalence regions, it is critical to establish the other factors associated with rotavirus in this post-vaccination period to improve other new preventive interventions against this viral infection. In addition, there are concerns about changes in the epidemiology of other prevalent viral infections, such as adenovirus, following the introduction of the Rotasiil^®^ vaccine.

In light of the preceding, this study aimed to determine the prevalence of rotavirus and adenovirus infections in children under five years of age with acute gastroenteritis and to identify factors associated with rotavirus infection after introducing the Rotasiil^®^ vaccine in 2019 in Kisangani, DRC.

## Materials and methods

### Study design and setting

Following the Strengthening the Reporting of Observational Studies in Epidemiology (STROBE) guidelines for reporting observational research [13], this study consisted of a cross-sectional and a hospital-based survey conducted from May 2022 to April 2023 in four health facilities (*Cliniques Universitaires de Kiangani*, *Hôpital Général de Référence de Makiso*, *Centre de Santé ALABUL*, and *Centre de Santé Nouveau Village de Pédiatrie*) in Kisangani, DRC, using a fecal-based test (BIOSYNEX Adenovirus/Rotavirus BSS, Biosynex SA, Illkirch-Graffenstaden, France) of rotavirus and adenovirus infections among children under five years with acute gastroenteritis.

### Study population and sample size

The survey targeted outpatients and inpatients under five years of age who sought medical attention at the four health facilities during the study period. All participants were volunteers recruited based on the following criteria: 1) are under five years old, 2) are experiencing acute gastroenteritis, and 3) have a mother’s consent to participate in the study. All neonates, children with chronic diarrhea (> 2 weeks), and those for whom maternal consent was not obtained were excluded from the study. Moreover, all patients with a positive malaria diagnosis were excluded from the final analysis due to the various possible biases they could generate.

The minimum sample size was 316 using the following formula: *n* = P(1–P)*(Z_α_)^2^ /i^2^ where P corresponded to the prevalence of rotavirus at 29% according to the previous study [14], and Z was 1.96 corresponding to the precision estimated at 5%.

### Data collection and procedure

Investigators (physicians) in each facility were previously trained in the protocol and how to collect samples. All data were prospectively collected using a pre-designed form. During hospital visits for medical consultations for outpatients and during medical ward rounds for inpatients, mothers of eligible patients for the study were given a brief explanation of the study’s purpose. After signing the informed consent form, the children, accompanied by their mothers, were admitted to a private setting for the study.

The physicians recorded the patients’ sex and clinical information about the appearance of diarrhea, level of dehydration, and body temperature based on the investigators’ observations. The mothers reported the sociodemographic information concerning the children’s age in years or months for those with less than one year, the mother’s education level, marital status and occupation, and the children’s clinical information such as vaccination status, dietary habits, and record of vomiting, fever, cough, and anorexia. All data were captured in the questionnaire. The rotavirus vaccination status was verified from the child’s vaccination card. Finally, the mothers were asked to complete a mini-exit questionnaire that assessed their knowledge of rotavirus vaccination using the Rotasiil^®^ vaccine. The questionnaire was structured, translated into three languages (French, Lingala, and Swahili), and self-administed. The questionnaire consisted of three questions: (1) knowledge of the rotavirus vaccine, (2) the need for three doses of the vaccine, and (3) the fact that the rotavirus vaccine does not prevent gastroenteritis of other causes in children.

Next, capillary blood was collected by finger pricking for malaria diagnosis using a WHO Prequalified Rapid Test for *P*. *falciparum* testing (ParaHIT^®^—*f* Ver 1.0 [Device], Arkray Healthcare Pvt. Ltd., Sachin [Surat], India). Approximately 10 ml of fecal material was collected in a 20 ml Falcon tube for rotavirus and adenovirus diagnosis using BYOSYNEX adenovirus/rotavirus BSS (Biosynex SA, Illkirch-Graffenstaden, France), a rapid in vitro diagnostic immunochromatographic test. As previously reported, only 50 μl of fecal material was used with the BYOSYNEX adenovirus/rotavirus BSS [15]. The remaining fecal samples were stored at -20°C for subsequent molecular analysis. All rapid test-handling procedures were conducted following the manufacturers’ recommendations.

### Outcomes

The primary study findings focused on rotavirus infections. Rotavirus infection was operationally defined as acute febrile or non-febrile gastroenteritis with a BIOSYNEX rapid test showing only two strips (T1: Rota and C: Control) and a negative malaria Rapid Diagnostic Test. The same definition was applied to adenovirus infections, but with a difference between the two BIOSYNEX test strips at position T2 (Adeno) and Control. Rotavirus and adenovirus coinfection was incriminated by the presence of three bands (T1, T2, and C) on the BIOSYNEX test [16]. Acute gastroenteritis is defined as passing loose or watery stools three or more times per day, with or without vomiting.

A rotavirus-vaccinated child was defined as a patient who had received at least one dose of the rotavirus vaccine. Children’s nutritional status was assessed and classified into three categories (malnutrition, normal nutritional status, and obesity) using the WHO weight-for-height score for children under five [17]. Mothers’ level of knowledge of rotavirus vaccination was considered high when they were aware of (1) the existence of Rotasiil^®^ as a rotavirus vaccine, (2) the need to administer all three doses in the vaccination schedule, and (3) the fact that the vaccine does not protect against other causes of gastroenteritis apart from rotavirus. The level of knowledge was average when mothers were aware of the vaccine and low when they were unaware of its existence.

### Statistical analysis

All data were recorded in an Excel database, and statistical analysis was conducted using R (version 4.2.1). First, descriptive statistics were computed using the mean (standard deviation) and proportion for quantitative and categorical data, respectively. The 95% CIs of the crude odds ratios (cOR) were calculated using Woolf’s logit method. Next, categorical data were compared using Pearson’s Chi-square test or Fisher’s exact test according to their validity criteria. A multivariate logistic regression using a stepwise model selection approach was conducted by integrating variables with a *P*-value < 0.1 obtained from Pearson’s Chi-square test, Fisher’s exact, or variables identified from the literature. The adjusted odds ratios (aOR) and their 95% CIs were analyzed to identify risk factors associated with rotavirus infection. Missing data (< 0.5%) were assigned the null value for conservative estimates; a *P*-value < 0.05 was considered statistically significant.

## Results

### Study population

**Fig 1:**
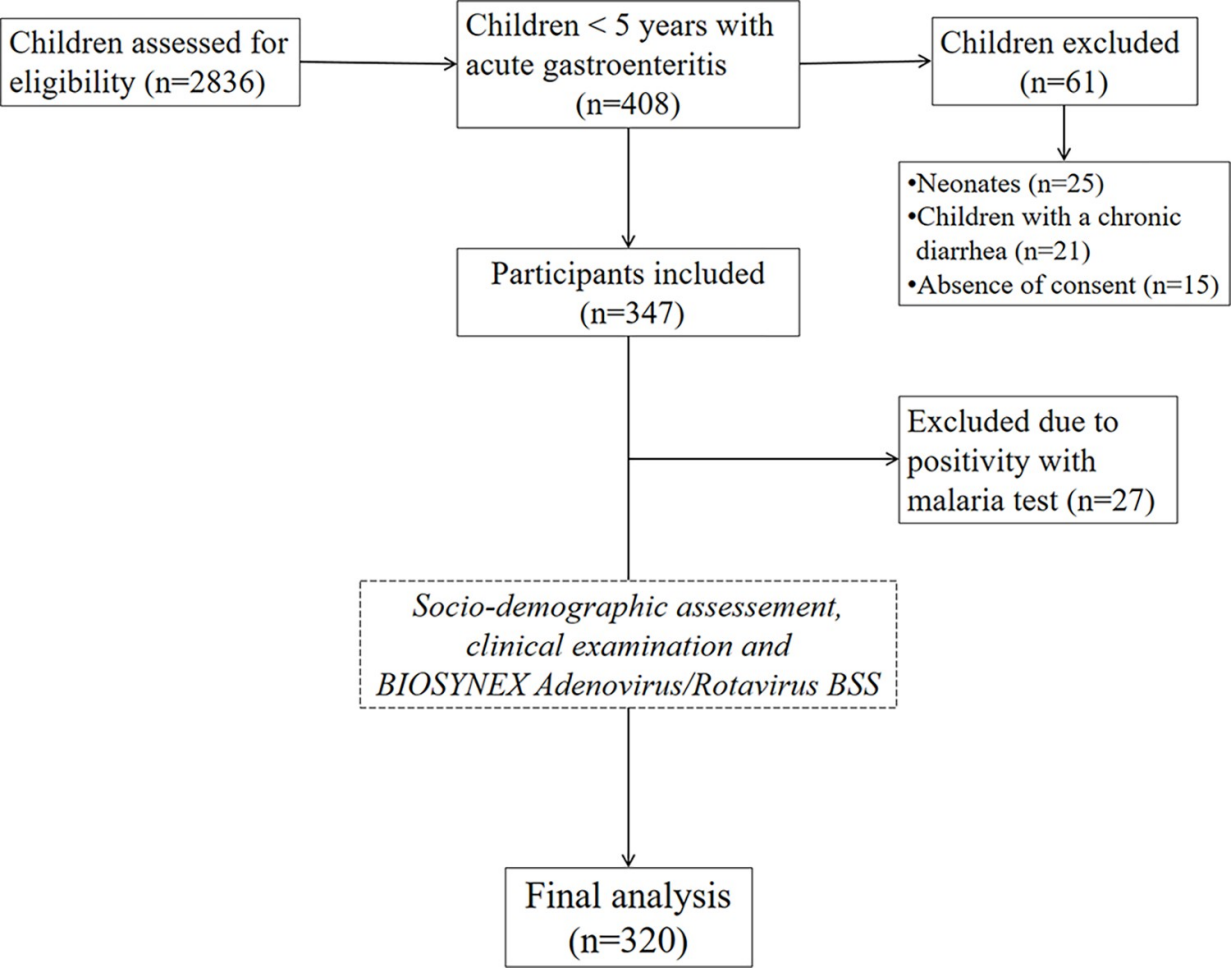
Flow chart showing the recruitment of study participants and their inclusion in the final analysis.

Fig 1 shows that 2,836 children were assessed for eligibility, including 408 children under five years of age with acute gastroenteritis. Among those under five years old with acute gastroenteritis, 347 were initially included in the study, whereas 61 were excluded due to: 1) age under 28 days of life (n = 25), 2) chronic diarrhea (n = 21), and 3) lack of children’s mothers’ consent (n = 15). Finally, 320 children under five years of age with acute gastroenteritis were included in the final analysis, excluding all participants with malaria (n = 27).

The sociodemographic and clinical characteristics of the study participants are shown in Table 1. Most participants were aged 1 to 11 months and of male gender; around two-thirds had been vaccinated against rotavirus, while less than half had received all three doses. Most children were fed a family meal and had a normal nutritional status. Most of the children’s mothers were married, had a secondary education, were unemployed, and had a low level of knowledge about the Rotasiil^®^ vaccine.

**Table 1:** Sociodemographic, behavioral, and clinical characteristics of 320 children under five years of age and their mothers participating in the study.

Characteristics	Number N = 320	Percent
**Age**		
1–5 months	72	22.5
6–11 months	122	38.1
1–2 years	71	22.2
> 2 –<5 years	55	17.2
**Gender**		
Female	113	35.3
Male	207	65.7
**Dietary habits**		
Mixed breastfeeding	22	6.9
Artificial milk	11	3.4
Breast milk	42	13.1
Family meal	245	76.6
**Nutritional status**		
Normal	296	92.5
Malnutrition	20	6.3
Overweight and obese	4	1.3
**Mother’s marital status**		
Single	32	10.0
Married	249	77.8
Partnered	39	12.2
**Mother’s occupation**		
With employment	106	33.1
Unemployed	198	61.9
Student	16	5.0
**Mother’s level of education**		
Primary	2	0.6
Secondary	173	54.1
University	145	45.3
**Mothers’ level of knowledge about the vaccine**		
Low level	198	61.9
Average level	63	19.7
High level	59	18.4
**Rotasiil**^**®**^ **vaccinated**		
No	112	35.0
Yes	208	65.0
**Number of Rotasiil**^**®**^ **vaccine doses**		
1	42	13.1
2	36	11.3
3	130	40.6
**Aspects of diarrhea**		
Mucous	146	45.6
Aqueous	170	53.1
Bloody	4	1.3
**Vomiting–**yes	235	73.4
**Fever–**yes	240	75
**Cough–**yes	167	52.2
**Abdominal pain–**yes	168	52.5
**Asthenia is defined by a refusal to play–**yes	244	76.2
**Anorexia–**yes	243	75.9

### Prevalence of rotavirus and adenovirus infections

**Fig 2:**
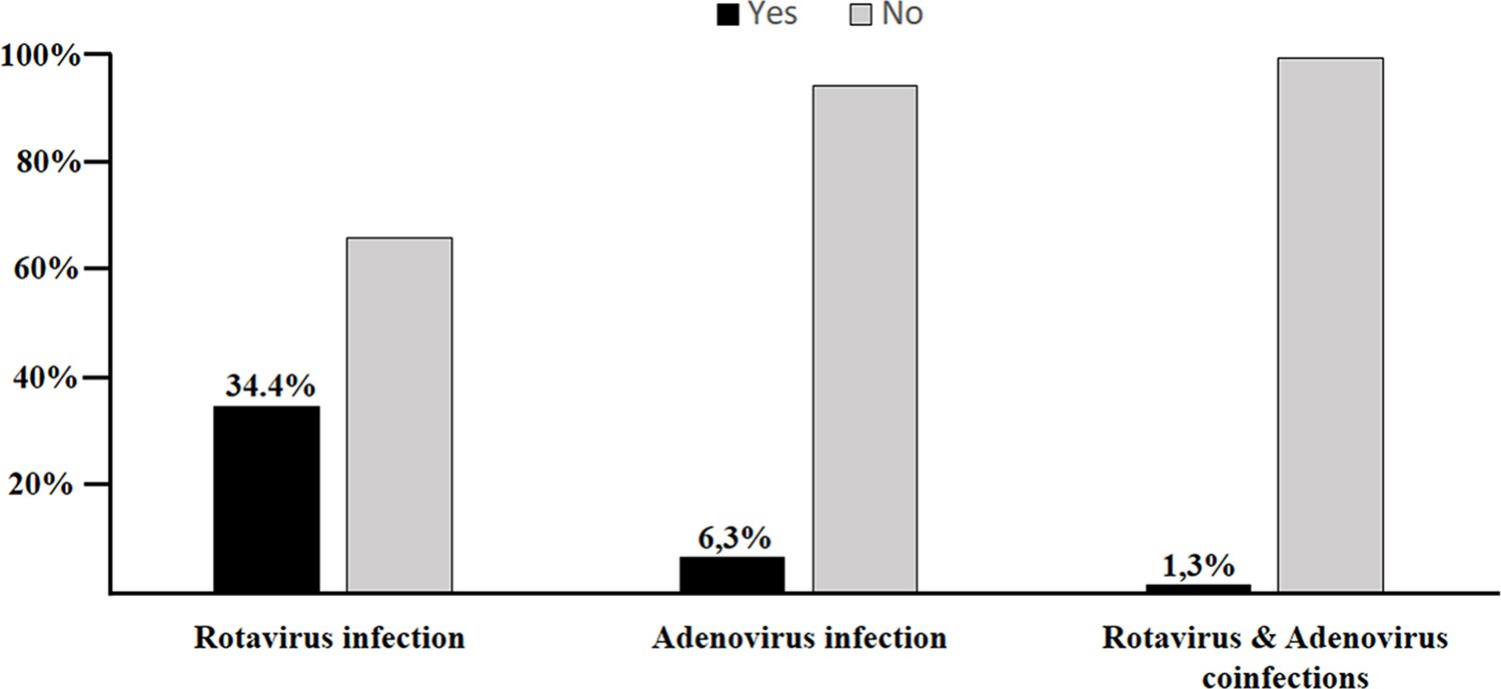
Prevalence of rotavirus and adenovirus and coinfections in children with acute gastroenteritis.

Overall, among the 320 participants in our series, 110 were positive for rotavirus alone, 20 for adenovirus alone, and four for both. These findings reflected a prevalence of rotavirus, adenovirus, and adenovirus/rotavirus coinfection of 34.4%, 6.3%, and 1.3%, respectively, as shown in Fig 2. Furthermore, among rotaviruspositive children, only 48 were vaccinated, whereas among negative children, 160 were vaccinated, giving low vaccination coverage (43.6%) among positive children versus high vaccination coverage (76.2%) among negative children.

**Fig 3:**
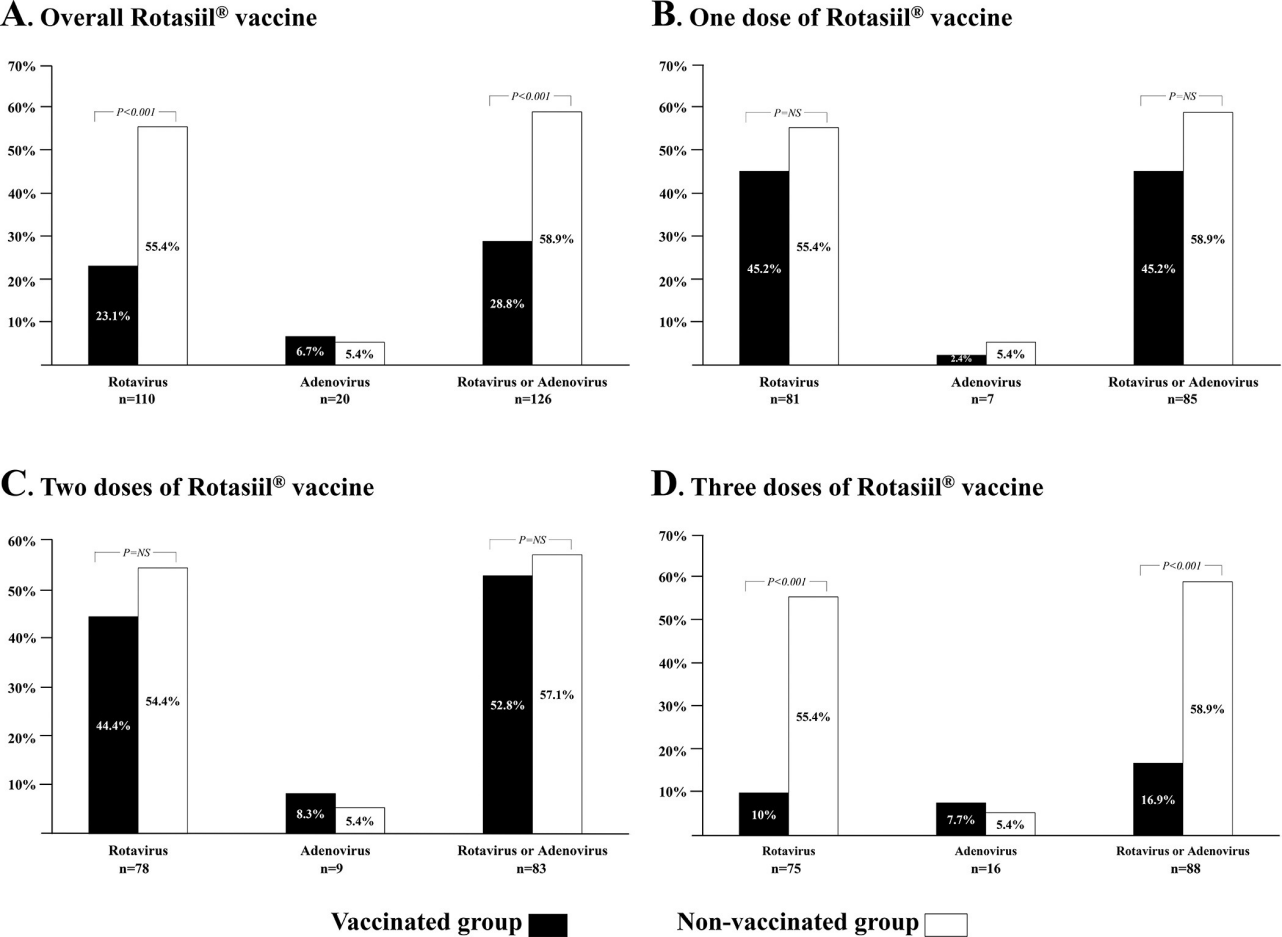
Bar chart comparing the percentages of rotavirus, adenovirus, and viral infection in children under five years of age with gastroenteritis, categorized into the Rotasiil-vaccinated and non-vaccinated groups. These comparisons were made overall (**A**) and according to the number of doses received, as follows: children who had received only one dose of vaccine (**B**), those who had received two doses (**C**), and those who had received three total doses (**D**).

As shown in Fig 3, the prevalence of rotavirus was significantly higher in unvaccinated children than in vaccinated children (55.4% versus 23.1%; *P* < 0.001) (Fig 3A). This difference was only observed in children who received all three doses of vaccine (Fig 3D), whereas no difference was observed in children who received a single dose (Fig 3B) or two doses (Fig 3C).

### Factors associated with rotavirus infection

When analyzing the factors associated with rotavirus infection, in bivariate analyses, we found that in addition to Rotasiil^®^ vaccination and the doses of this vaccination (Fig 3), variables such as children’s nutritional status, their mothers’ marital status, their mothers’ occupation, and their mothers’ level of knowledge about rotavirus vaccination were associated with rotavirus infection in children. The multivariate logistic regression analysis shows that the rate of rotavirus infection was significantly reduced in vaccinated children (from 55.4% to 23.1%; adjusted OR: 0.31 [95% CI: 0.19–0.56]; *P* < 0.001), especially those who have received all three doses of vaccine (from 55.4% to 10%; adjusted OR: 0.15 [95% CI: 0.06–0.37]; *P* < 0.001) compared to unvaccined children and those whose mothers had an average level (from 43.4% to 25.4%; adjusted OR: 0.51 [95% CI: 0.25–0.91]; *P* = 0.018) or a high level (from 43.4% to 13.5%; adjusted OR: 0.34 [95% CI: 0.20–0.64]; *P* < 0.001) of knowledge about the rotavirus vaccine compared to those with a low level. However, these analyses showed no link between rotavirus infection, children’s nutritional status, or their mothers’ marital status or occupation (see Table 2).

**Table 2:** Bivariate and multivariate regression analysis of factors associated with rotavirus infection among 320 children under 5 with gastroenteritis and their mothers participating in this study.

Characteristics	Rotavirus infection			
	Yes N = 110 n (%)	cOR [95%CI]	aOR [95%CI]	P-value
**Vaccinated against rotavirus**				
No	62 (55.4)	1	1	-
Yes	48 (23.1)	0.24 [0.14–0.41]	0.31 [0.19–0.56]	< 0.001
**Number of vaccine doses**				
0	62 (55.4)	1	1	
1	19 (45.2)	0.67 [0.32–1.35]	0.66 [0.27–1.57]	0.281
2	16 44.4)	0.65 [0.30–1.37]	0.64 [0.25–1.58]	0.280
3	13 (10.0)	0.09 [0.05–0.17]	0.10 [0.04–0.20]	< 0.001
**Nutritional status**				
Normal	97 (32.8)	1	1	-
Malnutrition	11 (55.0)	1.51 [0.59–3.76]	2.42 [0.66–4.59]	0.256
Overweight and obese	2 (50.0)	1.23 [0.17–8.87]	2.11 [0.09–14.23]	0.876
**Mother’s marital status**				
Single	14 (43.8)	1	1	-
Married	84 (33.7)	0.65 [0.31–1.38]	0.88 [0.32–3.98]	0.704
Partnered	12 (30.8)	0.57 [0.21–1.51]	0.79 [0.11–3.01]	0.658
**Mother’s occupation**				
With employment	30 (28.3)	1	1	-
Unemployed	72 (36.4)	1.45 [0.87–2.42]	2.37 [0.67–4.66]	0.833
Student	8 (50.0)	7.6 [2.2–25.4]	3.11 [0.91–33.3]	0.094
**Mother’s level of knowledge about the vaccine**				
Low level	86 (43.4)	1	1	-
Average level	16 (25.4)	0.46 [0.26–0.83]	0.51 [0.25–0.91]	0.018
High level	8 (13.5)	0.21 [0.09–0.47]	0.34 [0.20–0.64]	< 0.001

aOR: Adjusted Odd Ratio; CI: Confidence Interval; cOR: Crude Odd Ratio.

## Discussion

Our findings focus on the prevalence of rotavirus and the factors associated with this viral infection after the introduction of Rotasiil^®^ in 2019 in Kisangani, DRC. In addition, we have reported results on the prevalence of adenovirus to verify whether the rotavirus vaccine response has had a "springboard effect" on other enteric viral infections. Overall, this study showed that the prevalence of rotavirus infection remains high in Kisangani despite vaccination. However, complete vaccination with three doses significantly reduced the rotavirus infection rate, and mothers’ knowledge about the rotavirus vaccine positively influenced the rate of rotavirus infection. Finally, our series showed a very low prevalence of adenovirus infection and rotavirus–adenovirus coinfection.

We observed that the prevalence of rotavirus infection in Kisangani after the introduction of the rotavirus vaccine is low compared with that for the DRC as a whole, estimated at 60% at sentinel rotavirus infection surveillance sites [11]; it remains almost identical to that found by Heylen before the introduction of the rotavirus vaccine in its study including two health facities included in this study (*Hôpital Général de Référence de Makiso* and *Centre de Santé Nouveau Village de Pédiatrie)* [14]. This could be explained by the fact that a high number of unusual genotypes and gene segments of animal origin, as demonstrated by Heylen’s study in symptomatic children, were not part of the Rotasiil^®^ vaccine antigens. Furthermore, children unvaccinated against rotavirus (35% of children with gastroenteritis) could explain the slight decrease in the frequency of rotavirus infection after introducing the rotavirus vaccine.

Concerning the prevalence of adenovirus infection, we observed that it was 6.3% in children under five years of age with acute gastroenteritis, which was close to the prevalence of 5.5% found in Congo Brazzaville in children under five years of age hospitalized with acute gastroenteritis [18]. In Gabon, rotavirus was frequently detected in children under five with acute gastroenteritis, followed by adenovirus in a higher proportion than in our study [19]. In Lebanon, rotavirus was most frequently detected among children admitted with acute viral gastroenteritis (66.6%), followed by adenovirus and both rotavirus/adenovirus coinfection (8.4%). In contrast, our study found that the prevalence of coinfection was 1.3% [20].

As shown in this study, overall, the prevalence of rotavirus was significantly higher in unvaccinated children than in vaccinated children, demonstrating the importance of vaccination in preventing rotavirus infection. Some authors had observed, in a meta-analysis, a moderate association between the reduction in the risk of rotavirus gastroenteritis and the Rotasiil^®^ vaccine, in contrast to other rotavirus vaccines [21]. Children who received three doses of Rotasiil^®^ had a reduced rate of rotavirus infection compared to children with one or two doses. In a systematic review of the effect of additional doses of rotavirus vaccine (RotaTeq) on the prevention of diarrhea in young children, administration of an additional dose of rotavirus vaccine was likely to improve the vaccine’s immune response [22].

In India, where rotavirus infection is a significant public health problem, as in the DRC, Raju demonstrated the need for high rotavirus vaccination coverage in children as early as possible to achieve better protection against rotavirus [23]. In the United States, Wang demonstrated that one dose of pentavalent vaccine was associated with 88% efficacy against rotavirus gastroenteritis hospitalizations and emergency room visits, and 44% against all-cause gastroenteritis hospitalizations and emergency room visits. At the same time, a two-dose regimen increased the protective efficacy against rotavirus gastroenteritis hospitalizations to 94% [24]. We think that our results have provided additional information on the importance of full vaccination coverage with three doses of the Rotasiil^®^ vaccine to reinforce protection against rotavirus infection, even though this vaccination coverage may depend on the children’s mothers’ knowledge of the vaccine and the vaccination schedule, and the availability of the vaccine at vaccination centers.

In analyzing the factors associated with rotavirus infection in this study, logistic regression analysis shows that the rate of rotavirus infection was significantly reduced in children whose mothers had an average of knowledge about the rotavirus vaccine. However, these analyses showed no link between rotavirus infection and children’s nutritional status or their mothers’ marital status or occupation. In Uganda, Nakawesi demonstrated that no significant association was found between rotavirus infection and the child’s nutritional status; however, the association between the mother’s level of knowledge about vaccination and rotavirus infection was not assessed, although it was found that mothers with secondary and higher education had a statistically significant [OR 1.8; 95% CI 1.1–2.7] association with rotavirus [25]. In an analysis of response factors to rotavirus vaccination counseling in a private pediatric clinic in Malaysia, Kutty demonstrated that the level of awareness or knowledge of rotavirus disease and the rotavirus vaccine was significantly associated with vaccine acceptance [26].

This study may help in the effort to strengthen health education and counseling of children’s mothers regarding rotavirus vaccination. Counseling on rotavirus vaccination should begin early in the prenatal period to allow complete vaccination with three doses within the first three months of birth for better protection against rotavirus infection.

### Strengths and limitations

The strength of this study is that it presents, to our knowledge, for the first time the prevalence of rotavirus and adenovirus infections in children with acute gastroenteritis after introducing the Rotasiil^®^ vaccine in Kisangani, DRC, and the factors associated with rotavirus infection. However, there are some limitations in this study. First, gaps in the accurate diagnosis of rotavirose and adenovirose using immunochromatographic tests could lead to a risk of classification bias. Next, the fact that the hospital-based study is limited to patients who come to the pre-selected health facilities could lead to selection bias and impact on prevalence results, requiring a representative sampling of the target population.

### Conclusions

The prevalence of rotavirus infection remains high in Kisangani despite vaccination. However, the prevalence of adenovirus infections was low in our series. Complete vaccination with three doses and mothers’ average and high level of knowledge about the rotavirus vaccine significantly reduces the rate of rotavirus infection. It is, therefore, essential to strengthen the mothers’ health education, continue with the Rotasiil^®^ vaccine, and ensure epidemiological surveillance of rotavirus infection. In perspective, subsequent clinical trials carried out in the same biotope as this study could shed much more light on Rotasiil’s vaccine effectiveness in Kisangani.

## Supporting information

S1 Appendix Raw data. (XLSX)

## References

[1] Qazi S, Aboubaker S, MacLean R, Fontaine O, Mantel C, Goodman T, et al. Ending preventable child deaths from pneumonia and diarrhoea by 2025. Development of the integrated Global Action Plan for the Prevention and Control of Pneumonia and Diarrhoea. Arch Dis Child. 2015 Feb;100 Suppl 1:S23–8. doi: 10.1136/archdischild-2013-305429 25613963

[2] Walker CLF, Rudan I, Liu L, Nair H, Theodoratou E, Bhutta ZA, et al. Global burden of childhood pneumonia and diarrhoea. Lancet. 2013 Apr 20;381(9875):1405–1416. doi: 10.1016/S0140-6736(13)60222-6 23582727 PMC7159282

[3] Gladstone BP et al. Protective effect of natural rotavirus infection in an Indian birth cohort. New Engl J Med. 2011;365:337–346. doi: 10.1056/NEJMoa1006261 21793745 PMC3596855

[4] Clark A et al. Global Rotavirus Surveillance Network. Estimating global, regional and national rotavirus deaths in children aged <5 years: current approaches, new analyses and proposed improvements. PLoS One. 2017;12(9):e0183392. 28892480 10.1371/journal.pone.0183392 PMC5593200

[5] World Health Organization (WHO). Weekly epidemiological record. Rotavirus vaccines: WHOposition paper. Available from https://apps.who.int/iris/bitstream/handle/10665/342904/WER9628-eng-fre.pdf Accessed August 19, 2023

[6] Parashar UD et al. Global illness and deaths caused by rotavirus disease in children. Emerg Infect Dis. 2003;9:565–572. 3 doi: 10.3201/eid0905.020562 12737740 PMC2972763

[7] Tate JE et al. Global, regional, and national estimates of rotavirus mortality in children <5 years of age, 2000–2013. Clin Infect Dis. 2016;62 Suppl 2:S96–S105. 27059362 10.1093/cid/civ1013 PMC11979873

[8] For the DRC, a new dose hope against rotavirus india made vaccine. Available from https://www.path.org/drc.newdosehopeagaintsrotavirusindiamadevaccine.accessed August 15, 2023

[9] Skansberg A, Sauer M, Tan M, Santosham M, Jennings MC. Product review of the rotavirus vaccines ROTASIIL, ROTAVAC, and Rotavin-M1. Hum Vaccin Immunother. 2021 Apr 3;17(4):1223–1234. doi: 10.1080/21645515.2020.1804245 33121329 PMC8018392

[10] Troeger C, Khalil IA, Rao PC, Cao S, Blacker BF, Ahmed T, et al. Rotavirus Vaccination and the Global Burden of Rotavirus Diarrhea Among Children Younger Than 5 Years. JAMA Pediatr. 2018 Oct 1;172(10):958–965. doi: 10.1001/jamapediatrics.2018.1960 30105384 PMC6233802

[11] Luhata Lungayo C, Burke RM, Cikomola A, Mukamba E, Burnett E, Tate JE, et al. Epidemiology and pre-vaccine burden of rotavirus diarrhea in Democratic Republic of Congo (DRC): Results of sentinel surveillance, 2009–2019. Vaccine. 2022 Sep 3:S0264-410X(22)01031-3. doi: 10.1016/j.vaccine.2022.08.041 36068112 PMC11494495

[12] Lamberti LM, Ashraf S, Walker CL, Black RE. A Systematic Review of the Effect of Rotavirus Vaccination on Diarrhea Outcomes Among Children Younger Than 5 Years. Pediatr Infect Dis J. 2016 Sep;35(9):992–8. doi: 10.1097/INF.0000000000001232 27254030

[13] Von Elm E, Altman DG, Egger M, Pocock SJ, Gotzsche PC, Vandenbroucke JP. Strengthening the Reporting of Observational Studies in Epidemiology (STROBE) statement: guidelines for reporting observational studies. BMJ. 2007; 335(7624): 806–8. doi: 10.1136/bmj.39335.541782.AD 17947786 PMC2034723

[14] Heylen E, Batoko Likele B, Zeller M, Stevens S, De Coster S, Conceição-Neto N, et al. Rotavirus surveillance in Kisangani, the Democratic Republic of the Congo, reveals a high number of unusual genotypes and gene segments of animal origin in non-vaccinated symptomatic children. PLoS One. 2014 Jun 26;9(6):e100953. doi: 10.1371/journal.pone.0100953 24968018 PMC4072759

[15] Gbebangi-Manzemu D, Kampunzu VM, Vanzwa HM, Mumbere M, Bukaka GM, Likele BB, et al. Clinical profile of children under 5 years of age with rotavirus diarrhoea in a hospital setting in Kisangani, DRC, after the introduction of the rotavirus vaccine, a cross-sectional study. BMC Pediatr. 2023 Apr 24;23(1):193. doi: 10.1186/s12887-023-04022-0 37095482 PMC10123467

[16] BIOSYNEX® Adenovirus/Rotavirus BSS. Available from https://rhogen.es/biosynex-adenovirus rotavirus-bss. Accessed 10 August 2023

[17] WHO. Classification of nutritional stutus of infants and children. Available from https://www.who.int/nutrition/publications/childgrowthstandards. Accessed 18 July 2023

[18] Mayindou G, Ngokana B, Sidibé A, Moundélé V, Koukouikila-Koussounda F, Christevy Vouvoungui J, et al. Molecular epidemiology and surveillance of circulating rotavirus and adenovirus in Congolese children with gastroenteritis. J Med Virol. 2016 Apr;88(4):596–605. doi: 10.1002/jmv.24382 26378607

[19] Lekana-Douki SE, Kombila-Koumavor C, Nkoghe D, Drosten C, Drexler JF, Leroy EM. Molecular epidemiology of enteric viruses and genotyping of rotavirus A, adenovirus and astrovirus among children under 5 years old in Gabon. Int J Infect Dis. 2015 May;34:90–5. doi: 10.1016/j.ijid.2015.03.009 25796432

[20] Zaraket R, Salami A, Bahmad M, El Roz A, Khalaf B, Ghssein G, et al. Prevalence, risk factors, and clinical characteristics of rotavirus and adenovirus among Lebanese hospitalized children with acute gastroenteritis. Heliyon. 2020 Jun 20;6(6):e04248. doi: 10.1016/j.heliyon.2020.e04248 32613122 PMC7322251

[21] Sun ZW, Fu Y, Lu HL, Yang RX, Goyal H, et al. Association of Rotavirus Vaccines With Reduction in Rotavirus Gastroenteritis in Children Younger Than 5 Years: A Systematic Review and Meta-analysis of Randomized Clinical Trials and Observational Studies. JAMA Pediatr. 2021 Jul 1;175(7):e210347. doi: 10.1001/jamapediatrics.2021.0347 33970192 PMC8111566

[22] Middleton BF, Fathima P, Snelling TL, Morris P. Systematic review of the effect of additional doses of oral rotavirus vaccine on immunogenicity and reduction in diarrhoeal disease among young children. EClinicalMedicine. 2022 Oct 6;54:101687. doi: 10.1016/j.eclinm.2022.101687 36247922 PMC9561686

[23] Raju B, Parikh RP, Vetter VV, Kolhapure S. Epidemiology of rotavirus gastroenteritis and need of high rotavirus vaccine coverage with early completion of vaccination schedule for protection against rotavirus diarrhea in India: A narrative review. Indian J Public Health. 2019 Jul-Sep;63(3):243–250. doi: 10.4103/ijph.IJPH_307_18 31552856

[24] Wang FT, Mast TC, Glass RJ, Loughlin J, Seeger JD. Effectiveness of an incomplete RotaTeq (RV5) vaccination regimen in preventing rotavirus gastroenteritis in the United States. Pediatr Infect Dis J. 2013 Mar;32(3):278–83. doi: 10.1097/INF.0b013e318275328f 23014356

[25] Nakawesi JS, Wobudeya E, Ndeezi G, Mworozi EA, Tumwine JK. Prevalence and factors associated with rotavirus infection among children admitted with acute diarrhea in Uganda. BMC Pediatr. 2010 Sep 24;10:69. doi: 10.1186/1471-2431-10-69 20868488 PMC2955671

[26] Kannan Kutty P, Pathmanathan G, Salleh NM. Analysis of factors in response to rotavirus vaccination counselling in a private paediatric clinic. Med J Malaysia. 2010 Jun;65(2):127–32. 23756797

